# (2*R*,2′*S*)-2,2′-Bi­piperidine-1,1′-diium dibromide

**DOI:** 10.1107/S1600536813028754

**Published:** 2013-10-26

**Authors:** Guang Yang, Bruce C. Noll, Elena V. Rybak-Akimova

**Affiliations:** a Department of Chemistry, Tufts University, 62 Talbot Ave., Medford, MA 02155, USA; b Bruker AXS Inc., 5465 E. Cheryl Parkway, Madison, WI 53711, USA

## Abstract

The title compound, C_10_H_22_N_2_^2+^·2Br^−^, was synthesized *via* reduction of 2,2′-dipyridyl with Ni–Al alloy/KOH, followed by separation of diastereoisomers (*meso* and *rac*) by recrystallization from ethanol. Although the two bridging C atoms are optically active, these two chiral centers adopt an (*S*,*R*) configuration; thus, the title compound contains an achiral *meso* form of 2,2′-bi­piperidine. Both of the piperidinium rings adopt chair conformations, and the two N atoms are *trans* to each other; an inversion center is located in the mid-point of the central C—C bond. The conformation of the organic moiety resembles that of 1,1′-bi(cyclo­hexa­ne). The organic di­ammonium cations are linked to each other through hydrogen bonding with bromide counter-ions, each of which forms two hydrogen bonds (N—H⋯Br) with two adjacent organic cations, thus linking the latter together in sheets parallel to (100).

## Related literature

For more details about synthetic and characterization methods, see: Denmark *et al.* (2006 ▶); Herrmann *et al.* (2006 ▶). For the chemistry of related complexes, see: Mikhalyova *et al.* (2012 ▶); Lyakin *et al.* (2012 ▶). For a related structure (the racemic isomer of the title compound), see: Laars *et al.* (2011 ▶).
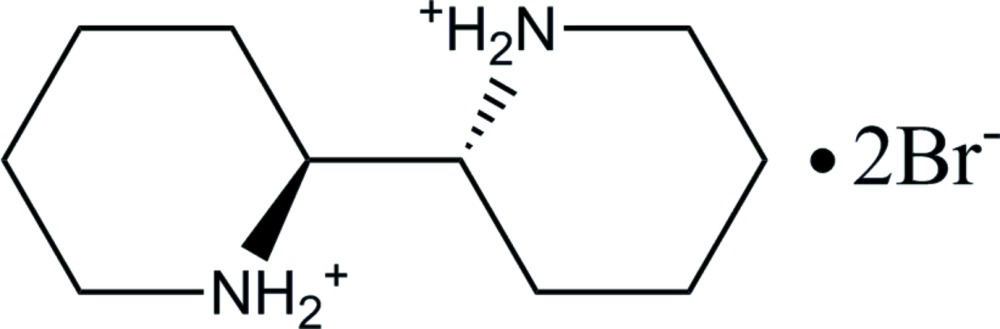


## Experimental

### 

#### Crystal data

C_10_H_22_N_2_^2+^·2Br^−^
*M* *_r_* = 330.11
Monoclinic, 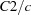

*a* = 17.9719 (9) Å
*b* = 9.7654 (5) Å
*c* = 7.3637 (3) Å
β = 92.134 (1)°
*V* = 1291.45 (11) Å^3^
*Z* = 4
Mo *K*α radiation
μ = 6.25 mm^−1^
*T* = 100 K
0.55 × 0.23 × 0.10 mm

#### Data collection

Bruker D8 QUEST CMOS diffractometer
Absorption correction: multi-scan (*SADABS*; Sheldrick, 2012 ▶) *T*_min_ = 0.13, *T*_max_ = 0.57
12426 measured reflections
2155 independent reflections
1955 reflections with *I* > 2σ(*I*)
*R*_int_ = 0.021

#### Refinement

*R*[*F* ^2^ > 2σ(*F* ^2^)] = 0.018
*wR*(*F* ^2^) = 0.045
*S* = 1.11
2155 reflections
70 parameters
H atoms treated by a mixture of independent and constrained refinement
Δρ_max_ = 0.88 e Å^−3^
Δρ_min_ = −0.24 e Å^−3^

### 

Data collection: *APEX2* and *Bruker Instrument Service* (Bruker, 2013 ▶); cell refinement: *SAINT* (Bruker, 2011 ▶); data reduction: *SAINT*; program(s) used to solve structure: *SHELXS97* (Sheldrick, 2008 ▶); program(s) used to refine structure: *SHELXL2013* (Sheldrick, 2008 ▶); molecular graphics: *OLEX2* (Dolomanov *et al.*, 2009 ▶); software used to prepare material for publication: *OLEX2*.

## Supplementary Material

Crystal structure: contains datablock(s) global, I. DOI: 10.1107/S1600536813028754/zl2557sup1.cif

Structure factors: contains datablock(s) I. DOI: 10.1107/S1600536813028754/zl2557Isup2.hkl

Additional supplementary materials:  crystallographic information; 3D view; checkCIF report

## Figures and Tables

**Table 1:** Hydrogen-bond geometry (Å, °)

*D*—H⋯*A*	*D*—H	H⋯*A*	*D*⋯*A*	*D*—H⋯*A*
N1—H1*A*⋯Br1^i^	0.913 (16)	2.408 (16)	3.2670 (10)	156.7 (13)
N1—H1*B*⋯Br1	0.880 (18)	2.359 (18)	3.2311 (10)	170.9 (14)

Symmetry code: (i) 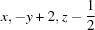.

**Fig. 1.**
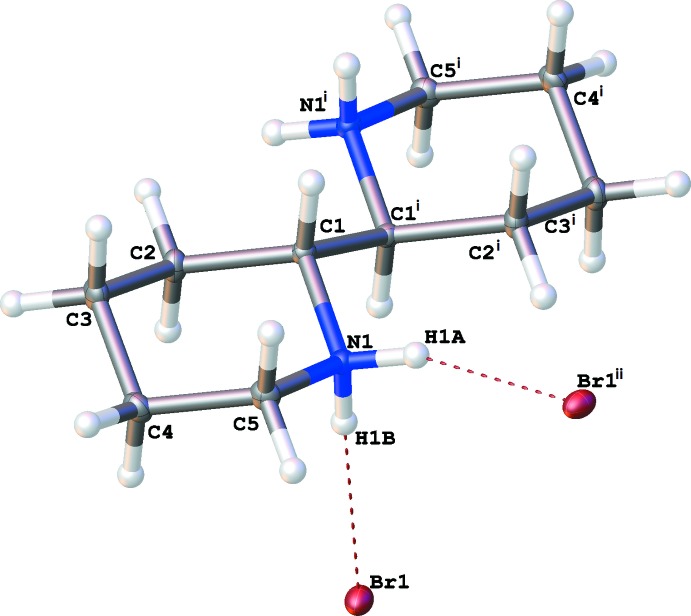
A view of the title compound, with displacement ellipsoids shown at the 50% probability level. Symmetry transformations used to generate equivalent atoms: (i) -x + 1/2, -y + 3/2, -z + 1.

**Fig. 2.**
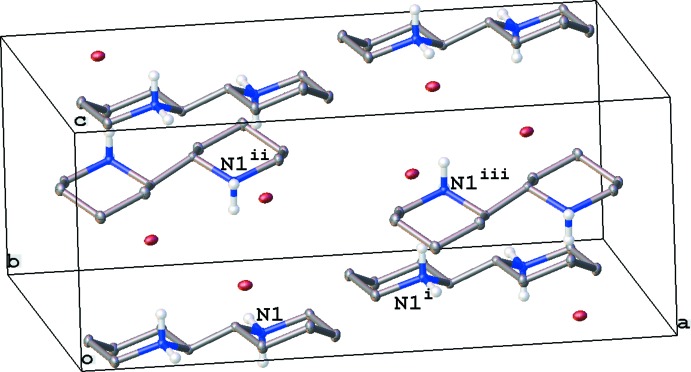
A fragment of the packing diagram of the title compound, with displacement ellipsoids shown at the 50% probability level (H atoms, except H atoms attached to N1 atom, are omitted for clarity). Symmetry transformations used to generate equivalent atoms: (i) -x + 1, -y + 1, -z; (ii) x, -y + 1, z + 1/2; (iii) -x + 1/2, y, -z + 1/2.

## supplementary crystallographic information

### 1. Comment

N-substituted 2-picolyl derivatives of the title compound, and related tetradentate aminopyridine ligands (Mikhalyova *et al.*, 2012; Lyakin *et al.*, 2012), in the form of Fe(II) and Mn(II) complexes, are good catalysts for selective olefin epoxidation, as well as aromatic or aliphatic hydroxylation reactions, with peroxides or peracids as oxidants. Rigid cyclic diamine scaffolds increase thermodynamic and kinetic stability of the catalysts and orient picolyl groups in the pseudo-octahedral complexes which also contain two labile solvent-occupied sites essential for hydrogen peroxide activation (Mikhalyova *et al.*, 2012). Understanding the molecular structure of these diamine scaffolds is important for catalyst design.

The title compound, which consists of organic diammonium cations and bromide counteranions (Figure 1), crystallizes in the monoclinic space group *C*2/*c*. The structure of the organic moiety resembles that of 1,1'-bi(cyclohexane), with both piperidinium rings adopting chair conformations. Two nitrogen atoms are *trans* to each other. The least square planes passing through the heavy (non-hydrogen) atoms of the two piperidinium rings in the centrosymmetric molecule are coplanar. This conformation differs from the twisted orientation of the piperidinium rings in the racemic isomer of the title compound (the angle between their least-square planes was found to be 77 °) (Laars *et al.*, 2011). Two stereocenters (bridging carbon atoms, C1 and C1') adopt different configurations (R and S, respectively) and are symmetry-related. The centrosymmetric *meso* form of C_10_H_22_N_2_Br_2_ described herein is achiral.

The organic diammonium cations are linked to each other through hydrogen bonding with bromide counter ions, each of which forms two hydrogen bonds (N—H···Br) with two adjacent organic cations, thus linking the latter together in sheets parallel to the (100) plane (Figure 2).

### 2. Experimental

The synthesis of the title compound followed the procedure published by Herrmann *et al.* (2006). 2,2'-Dipyridyl (8.25g, 0.053mol, in 100ml methanol) was mixed with 3M aqueous solution of KOH (16.8g, 0.3mol); Raney type Ni/Al alloy (1:1, v/v, 30g) was then added in portions and this mixture was stirred and refluxed for 4 days. The solution was separated from nickel mud, concentrated and extracted with 15ml dichloromethane (DCM) 3 times. The combined DCM extracts were evaporated to a faint yellow oil, which was then dissolved in 20ml ethanol. 9 ml of concentrated hydrobromic acid were added dropwise into the ethanolic solution; clear colorless hexagonal plate crystals of *meso*-C_10_H_22_N_2_Br_2_ were formed upon slow evaporation of the solution (one of these crystals was selected for X-Ray diffraction analysis).

Further evaporation (no less than 5ml) of the solvent liberated additional amount of *meso*-C_10_H_22_N_2_Br_2_. Yield: 6.2g (35%). More *meso*-C_10_H_22_N_2_Br_2 _was obtained by separation of *meso*- and *rac*-diastereomers of C_10_H_22_N_2_Br_2_ (from the remaining ethanolic solution) according to W.A. Herrmann *et al.* (2006) in 55% recovery. ^1^H NMR (500 MHz, D_2_O): δ 1.64 (m, 6, CH_2_), 1.96 (d, *J* = 14.5 Hz, 2, CH_2_), 2.02 (d, *J* = 10.5 Hz, 2, CH_2_), 2.14 (d, *J* = 11.5Hz, 2, CH_2_), 3.11 (t, *J* = 13.0 Hz, 2, CH_2_), 3.45 (d, *J* = 8.5 Hz, 2, CH), 3.56 (d, *J* = 12.5 Hz, 2, CH_2_). ^13^C NMR (500 MHz, D_2_O): δ 21.26, 21.49, 24.99, 46.02, 58.12. IR ν_max _/cm^-1^: 2907, 2714, 2390, 1573, 1413, 1358, 1307, 1010, 901, 605.

### 3. Refinement

Except where noted, hydrogen atoms were placed at calculated geometries and allowed to ride on the position of the parent atom. C–H distances were constrained to 1.00 Å for tertiary CH groups and to 0.99 Å for methylene groups (CH_2_). Isotropic thermal parameters were set to 1.2× the equivalent isotropic thermal parameter of the parent atom. Hydrogens H1A and H1B, bound to N1, were located in difference density Fourier synthesis maps. The positions of these two H atoms were freely refined. Isotropic thermal parameters for H1A and H1B were set to 1.2× the equivalent isotropic U of N1.

### Crystal data

**Table:**

C_10_H_22_N_2_^2+^·2Br^−^	*F*(000) = 664
*M**_r_* = 330.11	*D*_x_ = 1.698 Mg m^−^^3^
Monoclinic, *C*2/*c*	Mo *K*α radiation, λ = 0.71073 Å
*a* = 17.9719 (9) Å	Cell parameters from 8775 reflections
*b* = 9.7654 (5) Å	θ = 2.3–31.5°
*c* = 7.3637 (3) Å	µ = 6.25 mm^−^^1^
β = 92.134 (1)°	*T* = 100 K
*V* = 1291.45 (11) Å^3^	Plate, clear colorless
*Z* = 4	0.55 × 0.23 × 0.10 mm

### Data collection

**Table:**

Bruker D8 QUEST CMOS diffractometer	2155 independent reflections
Radiation source: sealed tube, Siemens KFFMO2K-90	1955 reflections with *I* > 2σ(*I*)
Curved graphite monochromator	*R*_int_ = 0.021
φ and ω scans	θ_max_ = 31.5°, θ_min_ = 2.3°
Absorption correction: multi-scan (*SADABS*; Sheldrick, 2012)	*h* = −26→26
*T*_min_ = 0.13, *T*_max_ = 0.57	*k* = −14→12
12426 measured reflections	*l* = −10→10

### Refinement

**Table:**

Refinement on *F*^2^	Primary atom site location: structure-invariant direct methods
Least-squares matrix: full	Secondary atom site location: difference Fourier map
*R*[*F*^2^ > 2σ(*F*^2^)] = 0.018	Hydrogen site location: mixed
*wR*(*F*^2^) = 0.045	H atoms treated by a mixture of independent and constrained refinement
*S* = 1.11	*w* = 1/[σ^2^(*F*_o_^2^) + (0.0237*P*)^2^ + 0.7137*P*] where *P* = (*F*_o_^2^ + 2*F*_c_^2^)/3
2155 reflections	(Δ/σ)_max_ = 0.001
70 parameters	Δρ_max_ = 0.88 e Å^−^^3^
0 restraints	Δρ_min_ = −0.24 e Å^−^^3^

### Special details

**Table:**

Geometry. All esds (except the esd in the dihedral angle between two l.s. planes) are estimated using the full covariance matrix. The cell esds are taken into account individually in the estimation of esds in distances, angles and torsion angles; correlations between esds in cell parameters are only used when they are defined by crystal symmetry. An approximate (isotropic) treatment of cell esds is used for estimating esds involving l.s. planes.
Refinement. Refinement of F^2^ against ALL reflections. The weighted R-factor wR and goodness of fit S are based on F^2^, conventional R-factors R are based on F, with F set to zero for negative F^2^. The threshold expression of F^2^ > 2sigma(F^2^) is used only for calculating R-factors(gt) etc. and is not relevant to the choice of reflections for refinement. R-factors based on F^2^ are statistically about twice as large as those based on F, and R- factors based on ALL data will be even larger.

### Fractional atomic coordinates and isotropic or equivalent isotropic displacement parameters (Å^2^)

**Table:**

	*x*	*y*	*z*	*U*_iso_*/*U*_eq_	
N1	0.15631 (5)	0.83374 (10)	0.52604 (14)	0.00987 (17)	
H1A	0.1668 (8)	0.9229 (17)	0.500 (2)	0.012*	
H1B	0.1566 (9)	0.8270 (15)	0.645 (2)	0.012*	
C1	0.21196 (6)	0.73540 (11)	0.45193 (15)	0.00905 (18)	
H1	0.2159	0.7519	0.3186	0.011*	
C2	0.18497 (6)	0.58942 (11)	0.48165 (17)	0.0123 (2)	
H2A	0.1854	0.5699	0.6136	0.015*	
H2B	0.2195	0.5245	0.4249	0.015*	
C3	0.10648 (6)	0.56754 (12)	0.40071 (17)	0.0136 (2)	
H3A	0.0903	0.4725	0.4243	0.016*	
H3B	0.1063	0.5813	0.2675	0.016*	
C4	0.05255 (6)	0.66795 (12)	0.48479 (17)	0.0133 (2)	
H4A	0.002	0.6545	0.4298	0.016*	
H4B	0.0505	0.6505	0.6169	0.016*	
C5	0.07765 (6)	0.81376 (12)	0.45314 (16)	0.0126 (2)	
H5A	0.0749	0.8341	0.3213	0.015*	
H5B	0.0441	0.8779	0.5144	0.015*	
Br1	0.14264 (2)	0.83601 (2)	0.96230 (2)	0.01383 (5)	

### Atomic displacement parameters (Å^2^)

**Table:**

	*U*^11^	*U*^22^	*U*^33^	*U*^12^	*U*^13^	*U*^23^
N1	0.0097 (4)	0.0102 (4)	0.0097 (4)	−0.0001 (3)	0.0005 (3)	−0.0007 (3)
C1	0.0087 (4)	0.0093 (4)	0.0092 (4)	0.0003 (4)	0.0004 (3)	−0.0005 (4)
C2	0.0110 (5)	0.0093 (5)	0.0165 (5)	−0.0010 (4)	−0.0010 (4)	0.0000 (4)
C3	0.0104 (5)	0.0128 (5)	0.0176 (5)	−0.0025 (4)	−0.0005 (4)	−0.0009 (4)
C4	0.0095 (5)	0.0147 (5)	0.0158 (5)	−0.0018 (4)	0.0016 (4)	0.0004 (4)
C5	0.0090 (5)	0.0142 (5)	0.0144 (5)	0.0008 (4)	−0.0006 (4)	0.0002 (4)
Br1	0.01971 (7)	0.01159 (7)	0.01027 (6)	−0.00222 (4)	0.00163 (4)	−0.00018 (4)

### Geometric parameters (Å, º)

**Table:**

N1—C1	1.5036 (14)	C2—H2B	0.99
N1—C5	1.5060 (15)	C3—C4	1.5260 (17)
N1—H1A	0.913 (16)	C3—H3A	0.99
N1—H1B	0.880 (18)	C3—H3B	0.99
C1—C2	1.5243 (16)	C4—C5	1.5143 (16)
C1—C1^i^	1.543 (2)	C4—H4A	0.99
C1—H1	1.0	C4—H4B	0.99
C2—C3	1.5259 (16)	C5—H5A	0.99
C2—H2A	0.99	C5—H5B	0.99
			
C1—N1—C5	114.59 (9)	C2—C3—C4	110.09 (9)
C1—N1—H1A	112.7 (10)	C2—C3—H3A	109.6
C5—N1—H1A	104.3 (10)	C4—C3—H3A	109.6
C1—N1—H1B	109.6 (10)	C2—C3—H3B	109.6
C5—N1—H1B	108.4 (11)	C4—C3—H3B	109.6
H1A—N1—H1B	106.8 (13)	H3A—C3—H3B	108.2
N1—C1—C2	109.01 (9)	C5—C4—C3	110.15 (9)
N1—C1—C1^i^	107.79 (11)	C5—C4—H4A	109.6
C2—C1—C1^i^	112.85 (11)	C3—C4—H4A	109.6
N1—C1—H1	109.0	C5—C4—H4B	109.6
C2—C1—H1	109.0	C3—C4—H4B	109.6
C1^i^—C1—H1	109.0	H4A—C4—H4B	108.1
C1—C2—C3	111.68 (9)	N1—C5—C4	110.36 (9)
C1—C2—H2A	109.3	N1—C5—H5A	109.6
C3—C2—H2A	109.3	C4—C5—H5A	109.6
C1—C2—H2B	109.3	N1—C5—H5B	109.6
C3—C2—H2B	109.3	C4—C5—H5B	109.6
H2A—C2—H2B	107.9	H5A—C5—H5B	108.1
			
C5—N1—C1—C2	−54.03 (12)	C1—C2—C3—C4	−58.25 (13)
C5—N1—C1—C1^i^	−176.84 (11)	C2—C3—C4—C5	58.08 (13)
N1—C1—C2—C3	54.76 (12)	C1—N1—C5—C4	55.44 (12)
C1^i^—C1—C2—C3	174.49 (11)	C3—C4—C5—N1	−55.86 (13)

Symmetry code: (i) −*x*+1/2, −*y*+3/2, −*z*+1.

### Hydrogen-bond geometry (Å, º)

**Table:**

*D*—H···*A*	*D*—H	H···*A*	*D*···*A*	*D*—H···*A*
N1—H1*A*···Br1^ii^	0.913 (16)	2.408 (16)	3.2670 (10)	156.7 (13)
N1—H1*B*···Br1	0.880 (18)	2.359 (18)	3.2311 (10)	170.9 (14)

Symmetry code: (ii) *x*, −*y*+2, *z*−1/2.

## References

[bb1] Bruker (2011). *SAINT* Bruker AXS Inc., Madison, Wisconsin, USA.

[bb2] Bruker (2013). *APEX2* and *Bruker Instrument Service* Bruker AXS Inc., Madison, Wisconsin, USA.

[bb3] Denmark, S. E., Fu, J. P. & Lawler, M. J. (2006). *J. Org. Chem.* **71**, 1523–1536. 10.1021/jo052203h 16468801

[bb4] Dolomanov, O. V., Bourhis, L. J., Gildea, R. J., Howard, J. A. K. & Puschmann, H. (2009). *J. Appl. Cryst.* **42**, 339–341.

[bb5] Herrmann, W. A., Baskakov, D., Herdtweck, E. D., Hoffmann, S. D., Bunlaksananusorn, T., Rampf, F. & Rodefeld, L. (2006). *Organometallics*, **25**, 2449–2456.

[bb6] Laars, M., Ausmees, K., Kudrjashova, M., Kanger, T. & Werner, F. (2011). *Acta Cryst.* E**67**, o1324. 10.1107/S1600536811016084 PMC3120339 21754721

[bb7] Lyakin, O. Y., Ottenbacher, R. V., Bryliakov, K. P. & Talsi, E. P. (2012). *ACS Catal.* **2**, 1196–1202.

[bb8] Mikhalyova, E. A., Makhlynets, O. V., Palluccio, T. D., Filatov, A. S. & Rybak- Akimova, E. V. (2012). *Chem. Commun.* **48**, 687–689. 10.1039/c1cc15935f 22134336

[bb9] Sheldrick, G. M. (2008). *Acta Cryst.* A**64**, 112–122. 10.1107/S0108767307043930 18156677

[bb10] Sheldrick, G. M. (2012). *SADABS* University of Göttingen, Germany.

